# Insights into the biological features and improved diagnostics of adult acute myeloid leukemia via fusion genes identified through targeted next-generation sequencing

**DOI:** 10.1016/j.cpt.2025.06.003

**Published:** 2025-06-10

**Authors:** Wei Guan, Ketao Wang, Yangliu Shao, Lei Zhou, Nan Wang, Wei Zhou, Maoquan Wang, Lili Wang, Yu Jing, Yonghui Li, Daihong Liu, Li Yu

**Affiliations:** a Department of Hematology, The Fifth Medical Center of Chinese PLA General Hospital, Beijing 100071, China; b Sports Medicine and Rehabilitation Department, Beijing Chaoyang Hospital, Capital Medical University, Beijing 100020, China; c Medical School of PLA Chinese PLA, Beijing 100853, China; d Central Laboratory, Shenzhen University General Hospital, Shenzhen University Medical School, Shenzhen, Guangdong 518060, China; e Department of Hematology-Oncology, International Cancer Center, Shenzhen University General Hospital, Shenzhen University Health Science Center, Shenzhen, Guangdong 518060, China

**Keywords:** Acute myeloid leukemia, Gene fusion, Next-generation sequencing, Diagnosis

## Abstract

**Background:**

Fusion genes play a crucial role in the pathogenesis of acute myeloid leukemia (AML). This study investigated the utility of targeted next-generation sequencing (NGS) of RNA for detecting rare and unknown fusion genes in patients with AML.

**Methods:**

A total of 85 adult AML samples previously identified as fusion gene-negative by multiplex nested reverse transcription-polymerase chain reaction (RT-PCR) were subjected to NGS analysis.

**Results:**

Fusion genes were detected in 21 of 72 (29.2%) patients. Among the 26 primary refractory patients, 11 (42.3%) exhibited fusion genes, whereas among the 18 relapsed patients, fusion genes were identified in five (27.8%). Notably, lysine methyltransferase 2A (*KMT2A*) and nucleoporin 98 (*NUP98*) rearrangements were enriched in refractory/relapsed patients. Additionally, recurrent fusion transcripts involving eukaryotic translation initiation factor 4A1 (*EIF4A1*) were identified. The identification of additional fusion genes resulted in an approximate 20.8% (11/53) reclassification of medium-risk karyotypes to the high-risk category, thereby enhancing diagnostic accuracy.

**Conclusions:**

Targeted NGS may complement conventional methods for identifying novel fusions in refractory/relapsed AML; however, its prognostic value requires validation in prospective controlled trials.

## Highlights

• Next-generation sequencing (NGS) identified fusion genes not identified by conventional polymerase chain reaction (PCR) methods, particularly in patients with refractory/relapsed acute myeloid leukemia (AML).
• Fusion genes were detected in 21 of the 72 cases (29.2%).
• Thus, approximately 20.8% (11/53) of medium-risk karyotypes were reclassified as high-risk, resulting in improved diagnostic accuracy.
• Newly identified fusion genes will enhance our understanding of this disease and facilitate stratification of prognosis.

## Graphical abstract

**Figure:**
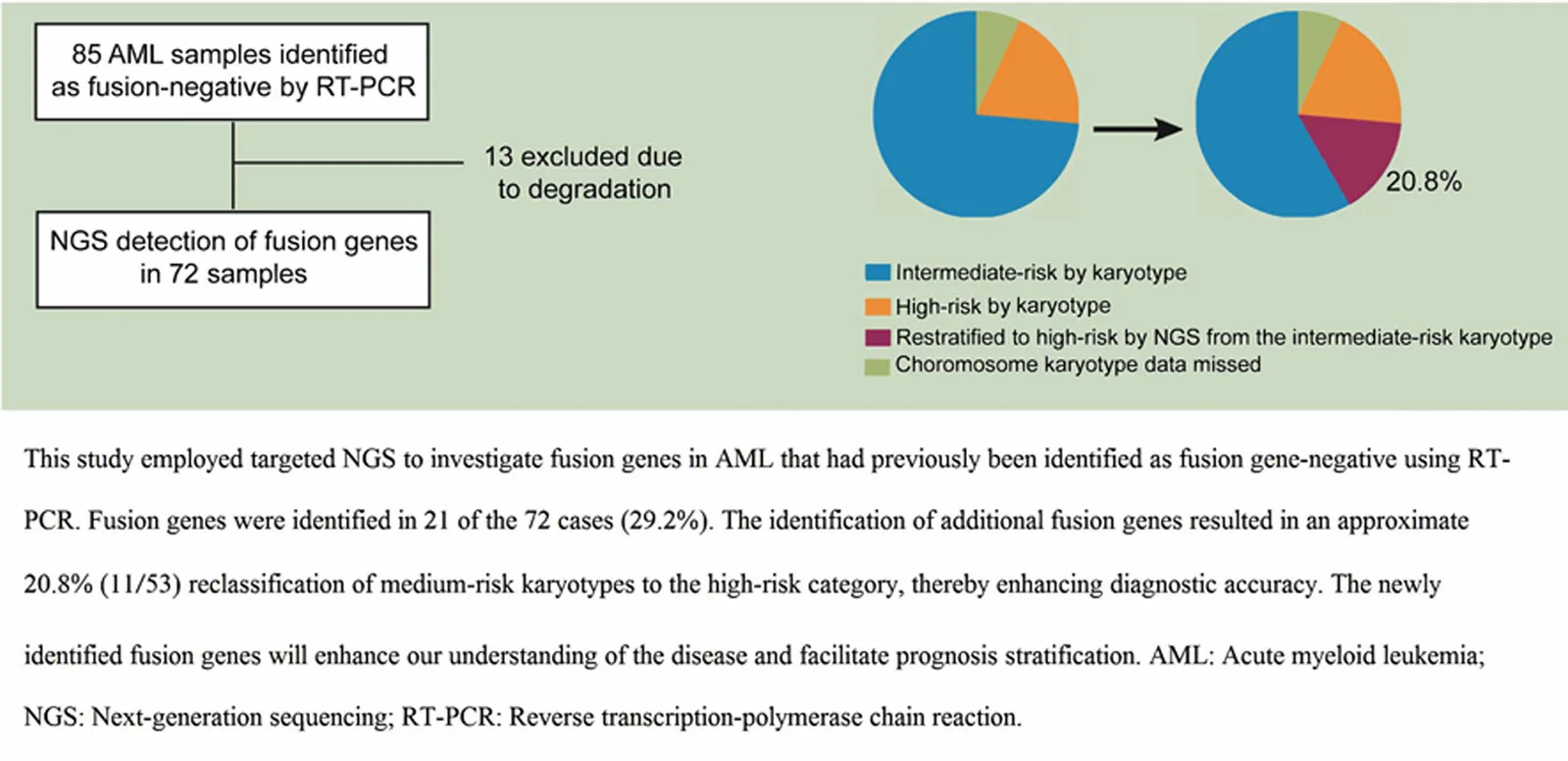

## Introduction

Acute myeloid leukemia (AML) is one of the most common malignant hematological malignancies and exhibits marked molecular and clinical heterogeneity. Approximately 40% of AML cases exhibit various clonal chromosomal aberrations.^1^ Hundreds of fusion genes have been identified, usually resulting from chromosomal structural aberrations such as chromosomal translocation, internal inversion, fragment deletion, or insertion and tandem duplication.^2^ However, the formation of fusion transcripts can also occur through transcription-mediated chimerism, which is detectable at the RNA rather than at the DNA level.^3^, ^4^, ^5^ The chimeric RNA Protein phosphatase 1 regulatory inhibitor subunit 1B-StAR related lipid transfer domain containing 3 (*PPP1R1B-STARD3*) is generated through post-transcriptional splicing of two adjacent genes, namely the upstream *PPP1R1B* and the downstream *STARD3*.^6^ The presence of gene fusions is associated with the leukemogenesis of AML and contributes to the heterogeneity of clinical characteristics and outcomes. The fusion proteins produced are highly biologically active because of the frequent involvement of proto-oncogenes, making them attractive targets for therapeutic interventions. Advanced technologies, such as next-generation sequencing (NGS), enable the comprehensive detection of fusion genes, thereby providing precise insights into their pathogenesis and offering prognostic evidence.

The pathogenic effects of these fusion genes render them ideal therapeutic targets. A prototypical example is the first identified fusion gene, breakpoint cluster region-abelson 1 (*BCR-ABL1*), which can trigger chronic myeloid leukemia (CML). Targeting *BCR-ABL1* with tyrosine kinase inhibitors (TKIs) has completely reversed the clinical prognosis of CML, transforming it into a manageable and curable chronic disease.^7^ Furthermore, fusion genes can serve as targets for monitoring measurable residual disease (MRD). MRD levels are closely associated with tumor burden, and stratified adjustments based on the MRD response after treatment can effectively reflect patient outcomes.^8^^,^^9^ Modifying the treatment regimen according to the MRD response helps to improve prognosis.

The current stratification of AML is based on cytogenetic and molecular genetic aberrations, categorizing patients into favorable-, intermediate-, and poor-prognosis groups. However, significant heterogeneity has been observed among patients with intermediate-risk AML.^10^ Some individuals within this group experience refractory or relapsed disease with a very poor prognosis, even in the absence of confirmed high-risk molecular factors. These high-risk subsets cannot be prospectively identified using conventional screening approaches.^11^ In previous studies, we identified that certain cases of AML exhibit rare or novel fusion genes, which are detected through panel NGS of RNA transcripts. These genes are frequently undetected during diagnosis using karyotyping and routine reverse transcription-polymerase chain reaction (RT-PCR).^12^ The presence of these undetected fusion genes may contribute to the heterogeneity observed in AML.

Based on this background, we employed targeted NGS to identify fusion genes in samples from 85 patients with AML, including newly diagnosed, relapsed, or refractory cases that had previously been classified as fusion-negative using conventional multiplex nested RT-PCR. The findings were evaluated in relation to the patients' clinical status. This study aimed to provide valuable insights into AML pathogenesis and enhance precise prognostic stratification.

## Materials and methods

### Patients and samples

This study used healthy adult donors as negative controls and 17 fusion gene-positive samples with hematological disorders as positive controls to confirm the reliability of the targeted NGS. The healthy donors included 10 males and 16 females, with a median age of 29 years (range: 25–37 years). All samples were obtained from peripheral blood. Positive control samples were mononuclear cells from bone marrow stored at −80 °C in TRIzol. Fusion gene detection was performed by two independent investigators blinded to clinical data. The median age of the patients was 34 years (range: 12–64 years), comprising four females and 13 males. The disease subtypes included AML (11 patients), lymphoma (three patients), CML (two patients), and multiple myeloma (MM) (one patient). The mean proportion of blast cells in the samples was 44% [Supplementary Table 1].

A total of 85 patients with AML who were negative for fusion gene detection using conventional RT-PCR and were admitted to our hospital between 2016 and 2017 were included. All the stored specimens were bone marrow mononuclear cells and were stored at −80 °C in TRIzol. Total RNA was extracted using a previously described protocol. Of the samples analyzed, 13 were degraded, while 72 were successfully constructed. The median age of the patients with detectable results was 50 years (range: 12–73 years), with a male-to-female ratio of 19:17. According to the French-American-British classification, the cases were classified as follows: M0 (*n*=2), M1 (*n*=2), M2 (*n*=15), M4 (*n*=19), M5 (*n*=27), and M6 (*n*=1), with 6 cases classified as information loss. The average proportion of blast cells was 56.6%. At the time of specimen collection, 44 (61.1%) patients were newly diagnosed with AML, 18 (25%) had relapsed AML, and 10 (13.9%) had refractory AML. This study did not include patients with low-risk karyotype profiles. There were 53 (73.6%) cases of medium-risk karyotypes, including 36 (50%) with normal karyotypes and 17 (23.6%) with abnormal chromosomes. Fourteen (19.4%) patients with high-risk karyotypes were identified. Karyotype information was missing in five (6.9%) cases [Table 1].

**Table 1:** General clinical characteristics of 72 patients with fusion gene-negative AML detected using RT-PCR.

Characteristics	Value
Age (years)	50 (12–73)
Male	38 (52.8)
FAB	
M0	2 (2.8)
M1	2 (2.8)
M2	15 (20.8)
M4	19 (26.4)
M5	27 (37.5)
M6	1 (1.4)
NA	6 (9.8)
Blasts in bone marrow (%)	61.8 (7.2–95.6)
WBC count at diagnosis (×10^9^/L)	35.9 (0.2–311.0)
Hemoglobin at diagnosis (g/L)	84.9 (30.0–148.0)
Platelet count at diagnosis (×10^9^/L)	84.4 (6.0–521.0)
Blasts in detected samples (%)	56.6 (4.0–95.6)
Status of patients enrolled	
Newly diagnosed	44 (61.1)
Relapsed	18 (25.0)
Primary refractory	10 (13.9)
Chromosome karyotype	
Low-risk	0
Intermediate-risk	53 (73.6)
Normal	37 (51.3)
Other	16 (22.2)
High-risk	14 (19.4)
NA	5 (6.9)

Data are presented as *n* (%) or median (range). AML: Acute myeloid leukemia; FAB: French-American-British; NA: Not applicable; RT-PCR: Reverse transcription-polymerase chain reaction; WBC: White blood cells.

### Multiplex nested polymerase chain reaction and targeted next-generation sequencing

At the time of diagnosis, all patients underwent bone marrow biopsy, and routine analyses included multiplex nested PCR to detect common fusion genes and chromosome karyotype analysis. The fusion gene panel included in the PCR covered the most common fusion genes in AML, as detailed in Supplementary Table 2. The multiplex nested PCR method is described in our previous study.^13^

NGS for fusion gene detection is based on targeted RNA sequencing (RNA-seq).^12^ In brief, RNA was extracted from patient samples using the Tempus Spin RNA Isolation Kit (Life Technologies, Carlsbad, CA, USA) following the manufacturer’s instructions. Samples comprising a total of 1500 ng RNA with an RNA integrity number ≥4.1 were used as the input for subsequent library preparation. Briefly, first- and second-strand complementary DNA (cDNA) synthesis was conducted using the PrimeScript™ Double Strand cDNA Synthesis Kit (Takara Bio Inc., Shiga, Japan). The double-stranded cDNA was then purified with Agencourt AMPure XP beads (Beckman Coulter, Inc., CA, USA), followed by end-repair, adenylation, and ligation using a universal barcode adapter, and subsequently amplified through seven cycles to generate mid-libraries. Target genes were captured from these mid-libraries using a specific panel, and were then amplified and sequenced. Paired-end, 101 bp sequencing was performed using a HiSeq 2500 (Illumina) instrument in Rapid Run mode. The sequences were aligned to the reference sequence using Hisat2 (2.0.3). FusionMap software was used to detect the fusion genes, and blacklist filtering was used to eliminate ribosomal genes, mitochondrial genes, and fusions involving pseudogenes, as well as fusions between gene families and homologous genes. The targeted fusion genes are listed in Supplementary Table 3.

### Statistical analysis

Data analysis and processing were conducted using EZR.R (version 1.53).^14^ The results are presented as mean values with their respective ranges. For the comparison of mean values between the two groups of independent samples, a *t*-test was used for data conforming to a normal distribution; otherwise, the non-parametric Wilcoxon signed-rank sum test was used. Categorical data are presented as frequencies and ranges, and the χ^2^ test was used for group comparisons.

## Results

### Negative and positive control cohort

Peripheral blood samples from 16 healthy adults were analyzed using NGS for fusion genes, and the results were all negative. The 17 identified samples with fusion genes included two cases of promyelocytic leukemia-retinoic acid receptor alpha (*PML-RARA*), three of RUNX family transcription factor 1- RUNX1 partner transcriptional co-repressor 1 (*RUNX1-RUNX1T1*), three of lysine methyltransferase 2A partial tandem duplication (*KMT2A-PTD*), one of lysine methyltransferase 2A-mixed-lineage leukemia translocated to 1 (*KMT2A-MLLT1*), three of MYC proto-oncogene-immunoglobulin heavy-chain (*MYC-IGH*), two of BCR activator of RhoGEF and GTPase-ABL proto-oncogene 1 (*BCR-ABL1*), one of cyclin D1-immunoglobulin heavy-chain (*CCND1-IGH*), and one of core-binding factor subunit beta-myosin heavy chain 1 (*CBFB-MYH1*). The fusion genes were confirmed using NGS, and the results were consistent with those of the RT-PCR analysis. NGS demonstrated a 100% concordance rate for the detection of fusion genes.

### Fusion genes detected by next-generation sequencing in reverse transcription-polymerase chain reaction-negative acute myeloid leukemia

**Figure 1:**
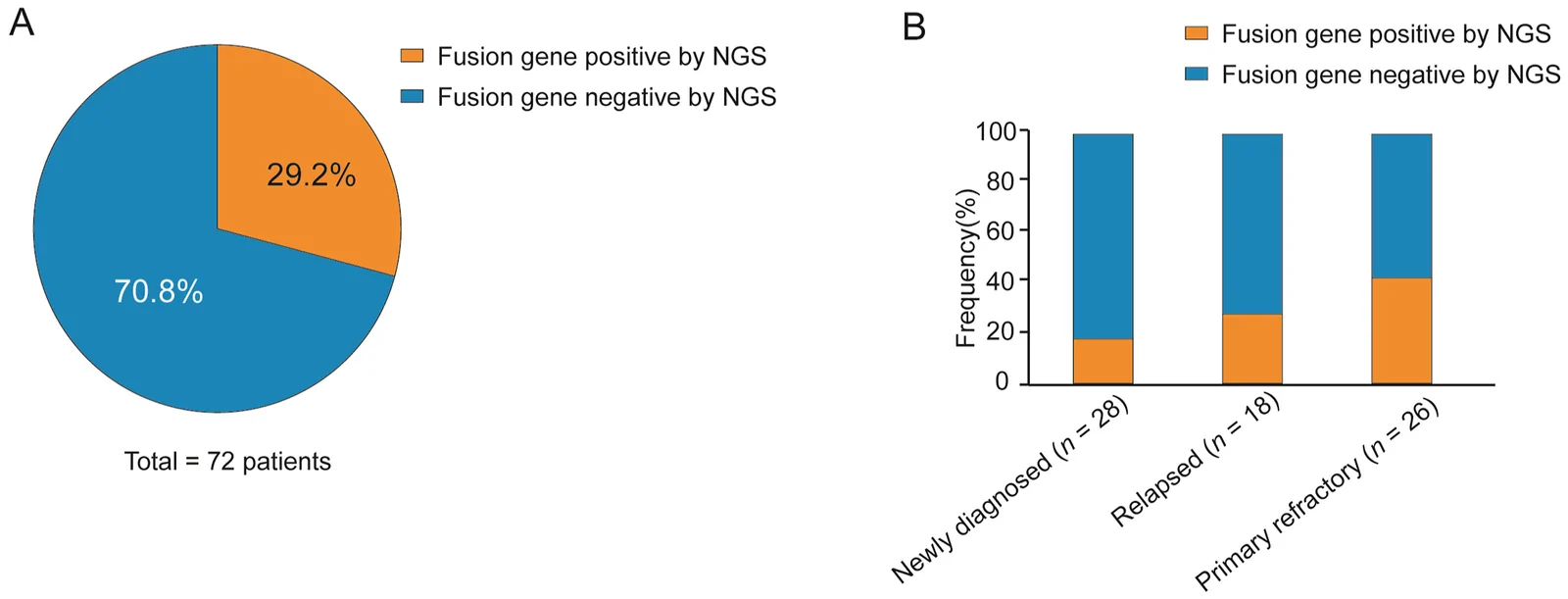
RT-PCR-negative specimens were re-examined using NGS to detect the presence of fusion genes. (A) Of the 72 RT-PCR-negative specimens, 21 (29.2%) were identified as carrying fusion genes after NGS. (B) Positive rate of fusion gene detection in the different groups. The positivity rate among newly diagnosed patients was 17.9% (5/28), 27.8% (5/18) in patients experiencing relapse, and 42.3% (11/26) in refractory patients. NGS: Next-generation sequencing; RT-PCR: Reverse transcription-polymerase chain reaction.

Among the 72 patients with successfully constructed samples who tested negative using conventional RT-PCR, fusion genes were detected in 21 (29.2%) using NGS [Figure 1A]. Eleven of the 26 primary refractory patients harbored fusion genes, resulting in a positive detection rate of 42.3%. Among the 18 patients with relapsed disease, fusion genes were detected in five, yielding a fusion gene positivity rate of 27.8%. In the group of newly diagnosed patients (*n* = 28), fusion genes were identified in five patients (17.9%). These newly diagnosed patients were stratified according to their subsequent treatment responses. In the non-relapse group (*n* = 11), three individuals tested positive for fusion gene expression, with a positivity rate of 27.3%. Among those who experienced relapse after remission (*n* = 13), two showed the presence of fusion genes, with a positivity rate of 15.4%. Notably, no fusion genes were detected in any patient who died during induction therapy (*n* = 4) [Figure 1B and Table 2]. The detection rate of fusion genes varied across different disease states, with the highest rate observed in patients with primary refractory disease.

**Table 2:** Fusion genes identified using NGS in different cohorts of patients with AML patients.

Group	*Value*	Fusion gene positive
Primary refractory	26 (36.1)	11 (42.3)
Relapse	18 (25.0)	5 (27.8)
Newly diagnosed	28	5 (17.9)
Relapse after remission	13	2 (15.4)
Non-relapse	11	3 (27.3)
Early death	4	0 (0)

Total	72	21 (29.1)

Data are presented as *n* or *n* (%). AML: Acute myeloid leukemia; NGS: Next-generation sequencing.

**Figure 2:**
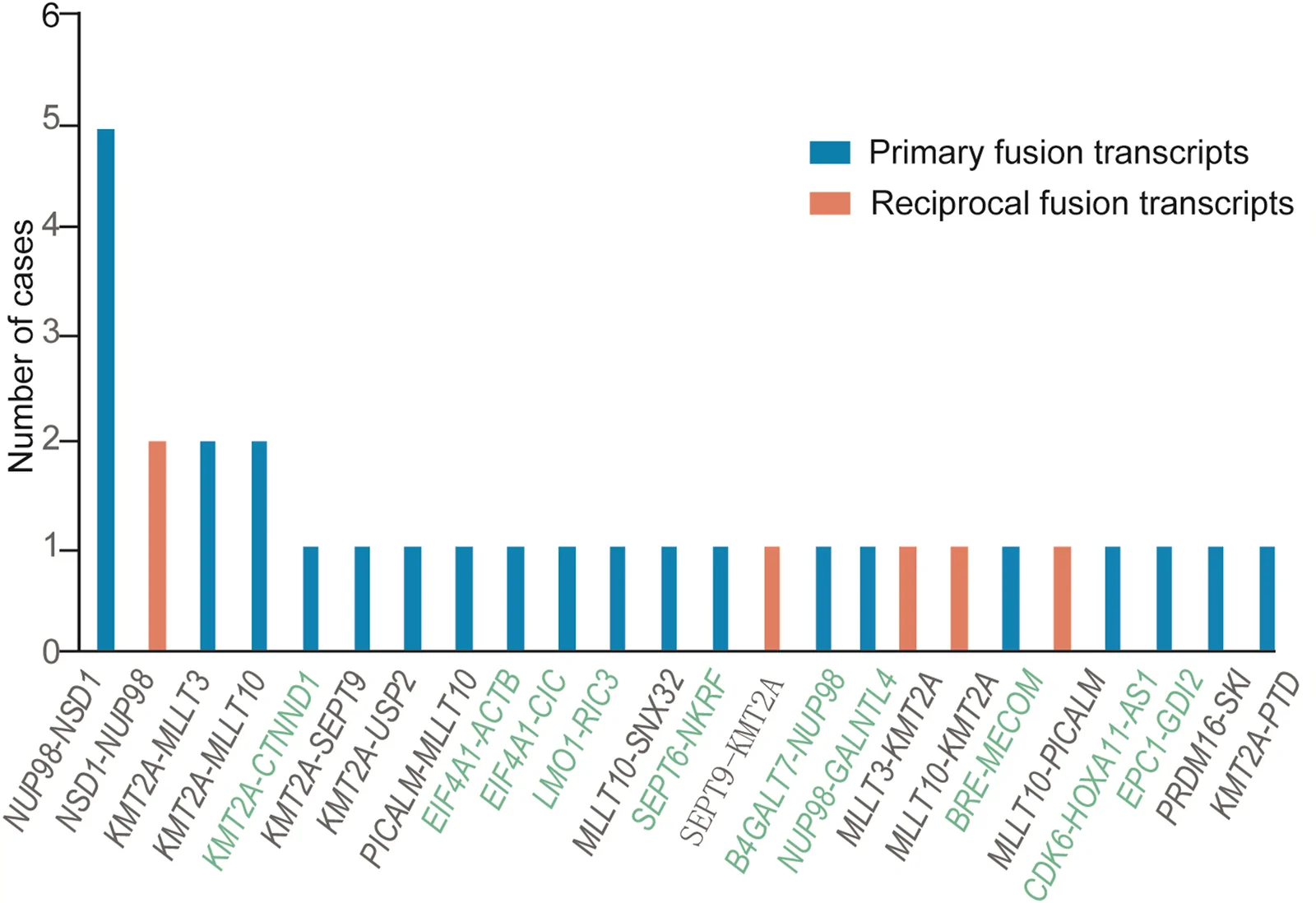
Fusion genes identified by targeted next-generation sequencing in routine RT-PCR-negative AML samples. The results include primary and reciprocal fusion transcripts, with novel fusion transcripts highlighted in green font. AML: Acute myeloid leukemia; *B4GALT7-NUP98:* Beta-1,4-galactosyltransferase 7-nucleoporin 98*;* BRE-MECOM: BRCA1-associated RING domain 1 enhancer-MDS1 and EVI1 complex locus*; CDK6-HOXA11-AS1:* Cyclin-dependent kinase 6- HOXA11 antisense RNA 1*; EIF4A1-CIC:* Eukaryotic translation initiation factor 4A1-capicua transcriptional repressor*; EPC1-GDI2:* Enhancer of polycomb homolog 1-GDP dissociation inhibitor 2; *KMT2A-CTNND1:* Lysine methyltransferase 2A-lysine methyltransferase 2A; *KMT2A-MLLT10:* Lysine methyltransferase 2A-MLLT10 histone lysine methyltransferase DOT1L cofactor; *KMT2A-MLLT3:* Lysine methyltransferase 2A-MLLT3 super elongation complex subunit; *KMT2A-SEPT9:* Lysine methyltransferase 2A-septin 9; *KMT2A-USP2:* Lysine methyltransferase 2A-ubiquitin-specific peptidase 2*; KMT2A-PTD:* Lysine methyltransferase 2A partial tandem duplication; *LMO1-RIC3:* LIM domain only 1- RIC3 chaperone; *MLLT10-KMT2A:* MLLT10 histone lysine methyltransferase DOT1L cofactor-lysine methyltransferase 2A; *MLLT10-PICALM:* MLLT10 histone lysine methyltransferase DOT1L cofactor-phosphatidylinositol binding clathrin assembly protein; *MLLT10*-*SNX32*: MLLT10 histone lysine methyltransferase DOT1L cofactor-sorting nexin 32; *MLLT3-KMT2A:* MLLT3 super elongation complex subunitlysine methyltransferase 2A; *NUP98-GALNTL4:* Nuclear receptor binding SET domain protein 1-polypeptide N-Acetylgalactosaminyltransferase-Like 4; *NUP98-NSD1:* Nucleoporin 98- Nuclear Receptor Binding SET Domain Protein 1; *NSD1-NUP98:* Nuclear receptor binding SET domain protein 1-nuclear receptor binding SET domain protein 1; *PICALM-MLLT10:* Phosphatidylinositol binding clathrin assembly protein-MLLT10 histone lysine methyltransferase DOT1L cofactor; *PRDM16-SKI:* PR/SET Domain 16-SKI proto-oncogene; RT-PCR: Reverse transcription-polymerase chain reaction; *SEPT6-NKRF:* Septin 6-NF-κB repressing factor; *SEPT9-KMT2A:* Septin 9-lysine methyltransferase 2A.

The gene distributions of the 21 identified positive fusion transcripts are shown in Table 2 and Figure 2. A total of 31 fusion gene forms were detected across the 21 samples, with each sample carrying an average of 1.5 fusion genes (range: 1–2). Among them, 11 samples (52.3%) exhibited only one type of fusion. In 10 samples (47.6%), two simultaneous forms of fusion genes were observed; six cases (28.6%) harbored complementary reciprocal fusion pairs derived from the same chromosomal translocation (e.g., *NUP98*-*NSD1* plus nucleoporin 98 And 96 precursor -nuclear receptor binding SET domain protein 1 [*NSD1*-*NUP98*]), whereas the remaining four cases (19.0%) exhibited two genetically distinct fusion events (e.g., *KMT2A*-catenin Delta 1 [*CTNND1*] co-occurring with Eukaryotic translation initiation factor 4A1-actin beta [*EIF4A1*-*ACTB*]).

### Fusion gene characteristics in different groups

**Figure 3:**
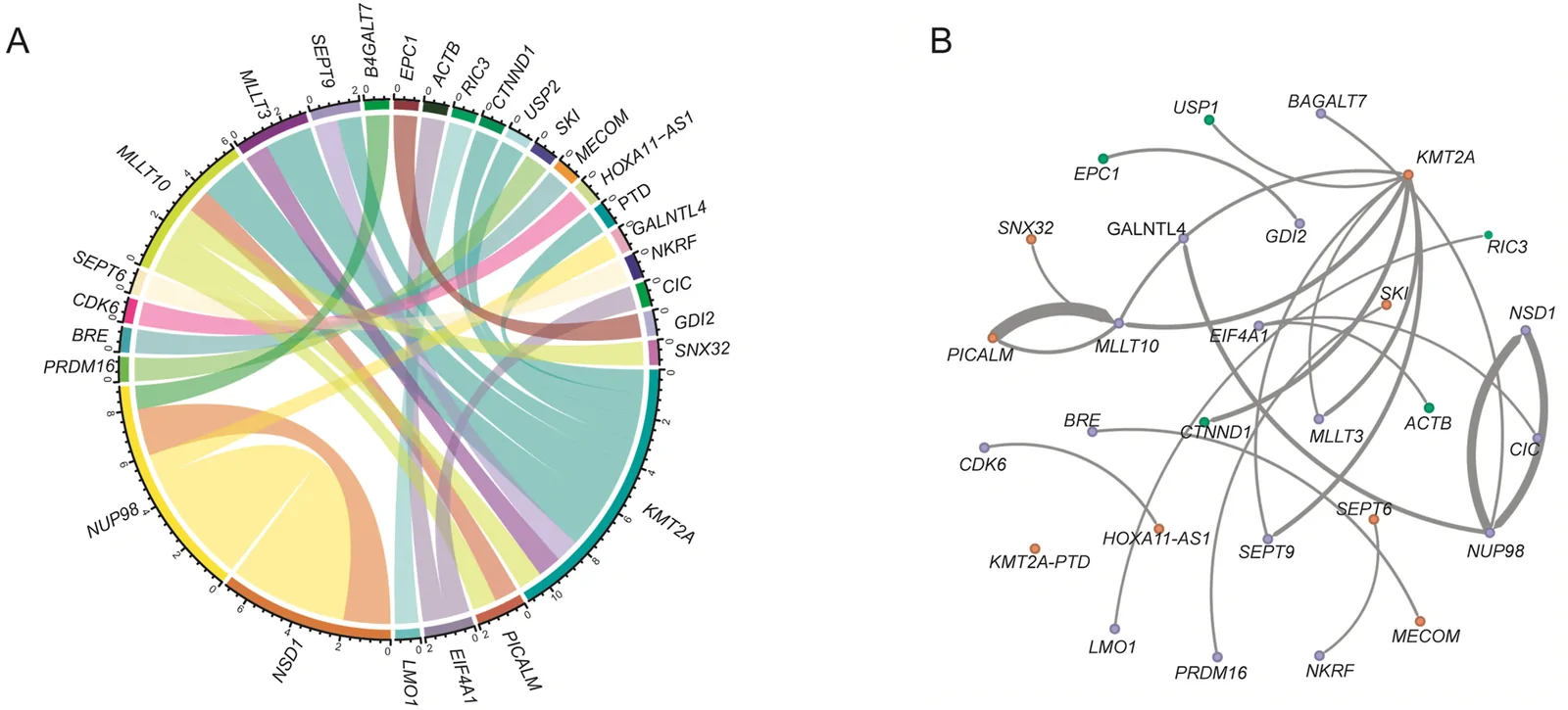
Fusion transcripts identified using next-generation sequencing from routine reverse transcription-polymerase chain reaction-negative acute myeloid leukemia samples. (A) The plot was generated using Circos. The connections between genes represent the occurrence of fusion genes, and the thickness of the lines represents the strength of the association. (B) Fusion gene network in this study. The lines represent fusions, and the thickness of the lines reflects the frequency of the observed fusions. *ACTB:* Actin beta*; B4GALT7:* Beta-1,4-galactosyltransferase 7*; BRE:* BRCA1-regulated enhancer*; CDK6:* Cyclin-dependent kinase 6*; CIC:* Capicua transcriptional repressor*; CTNND1:* Catenin delta 1*; EIF4A1:* Eukaryotic translation initiation factor 4A1*; EPC1:* Enhancer of polycomb homolog 1*; GALNTL4:* Polypeptide N-acetylgalactosaminyltransferase-like 4*; GDI2:* GDP dissociation inhibitor 2*; HOXA11-AS1:* HOXA11 antisense RNA 1*; KMT2A:* Lysine methyltransferase 2A*; KMT2A-PTD:* Lysine methyltransferase 2A partial tandem duplication*; LMO1:* LIM domain only 1*; MECOM:* MDS1 and EVI1 complex locus*; MLLT10:* MLLT10 histone lysine methyltransferase DOT1L cofactor*; MLLT3:* MLLT3 super elongation complex subunit*; NKRF:* NF-κB repressing factor*; NSD1: N*uclear receptor binding SET domain protein 1*; NUP98:* Nucleoporin 98*; PICALM:* Phosphatidylinositol binding clathrin assembly protein*; PRDM16:* PR/SET Domain 16*;* PTD: Partial tandem duplication; *RIC3:* RIC3 chaperone*; SEPT6:* Septin 6*; SEPT9:* Septin 9*; SKI:* SKI proto-oncogene*; SNX32:* Sorting Nexin 32*; USP1:* Ubiquitin-specific peptidase 1*; USP2:* Ubiquitin-specific peptidase 1.

Among the 26 primary refractory patients, a medium-risk karyotype was observed in 19 (73.1%), a high-risk karyotype in six (23.1%), and karyotype information was missing for one. The most frequently identified fusion genes were *KMT2A*-r (also known as *MLL*-r) and *NUP98*-r [Figure 3]. Specifically, four *KMT2A* fusion genes were identified: *KMT2A-CTNND1*, *KMT2A-MLLT3*, *KMT2A-septin 9 (SEPT9*), and *KMT2A-ubiquitin Specific Peptidase 2 (USP2*). Four of the five *NUP98* rearrangements were *NUP98-NSD1* rearrangements. The beta-1,4-galactosyltransferase 7 (*B4GALT7)-NUP98* fusion gene was detected in addition to *NUP98-NSD1* in patient No. YF00HFG00M000037. Interestingly, repeated changes involving *EIF4A1* were observed in two cases of *EIF4A1* rearrangements: *EIF4A1-ACTB* and *EIF4A1-CIC*. In addition, one patient exhibited a *PRDM16-SKI* fusion gene [Supplementary Table 4].

Among the four cases of *KMT2A* fusion, one exhibited the *KMT2A-MLLT3* fusion gene, which was associated with a medium-risk prognosis. Additionally, one patient with *KMT2A-CTNND1* fusion had a complex karyotype. The presence of an *EIF4A1* fusion gene has not been previously reported. However, despite their intermediate-risk karyotype, both patients with *EIF4A1* rearrangements exhibited primary drug resistance, suggesting that alterations in this gene may be significant risk factors. Notably, AML-associated fusions, including *EIF4A1-ACTB*, LIM domain only 1-RIC3 cholinergic receptor chaperone (*LMO1-RIC3*), *EIF4A1-CIC*, *B4GALT7-NUP98*, and *KMT2A-CTNND1* were reported for the first time.

Among the 18 patients with relapsed disease, fusion genes were detected in five individuals (27.8%). These included *KMT2A-PTD, brain and reproductive organ-expressed-MDS1 and EVI1 complex locus (BRE-MECOM), cyclin-dependent kinase 6 - HOXA11 antisense RNA 1(CDK6-HOXA11-AS1), septin 6-NF-κB repressing factor (SEPT6-NKRF*), and one patient who simultaneously expressed *NUP98-GALNTL4* and *EPC1-GDI2* fusion genes [Supplementary Table 4]. The identification of three novel fusion transcripts, namely *BRE-MECOM*, *CDK6-HOXA11-AS1*, and *SEPT6-NKRF*, represents a significant advancement in AML research.

Among the 13 patients who experienced relapse after remission, two (13.3%) carried the fusion gene at the time of diagnosis. One patient exhibited both *KMT2A-MLLT10* and *MLLT10-SNX32* fusions, whereas the other exhibited *KMT2A-MLLT3* fusion. In the non-relapse group (*n* = 11), fusion genes were detected in three patients, resulting in a positive detection rate of 27.3%. The identified fusion genes included one case each of Phosphatidylinositol binding clathrin assembly protein (*PICALM-MLLT1*), *NUP98-NSD1*, and *KMT2A-MLLT10*. The latter two patients underwent allogeneic hematopoietic stem cell transplantation during complete response and achieved continued remission thereafter.

### Re-stratification of intermediate-risk karyotype acute myeloid leukemia by next-generation sequencing

**Figure 4:**
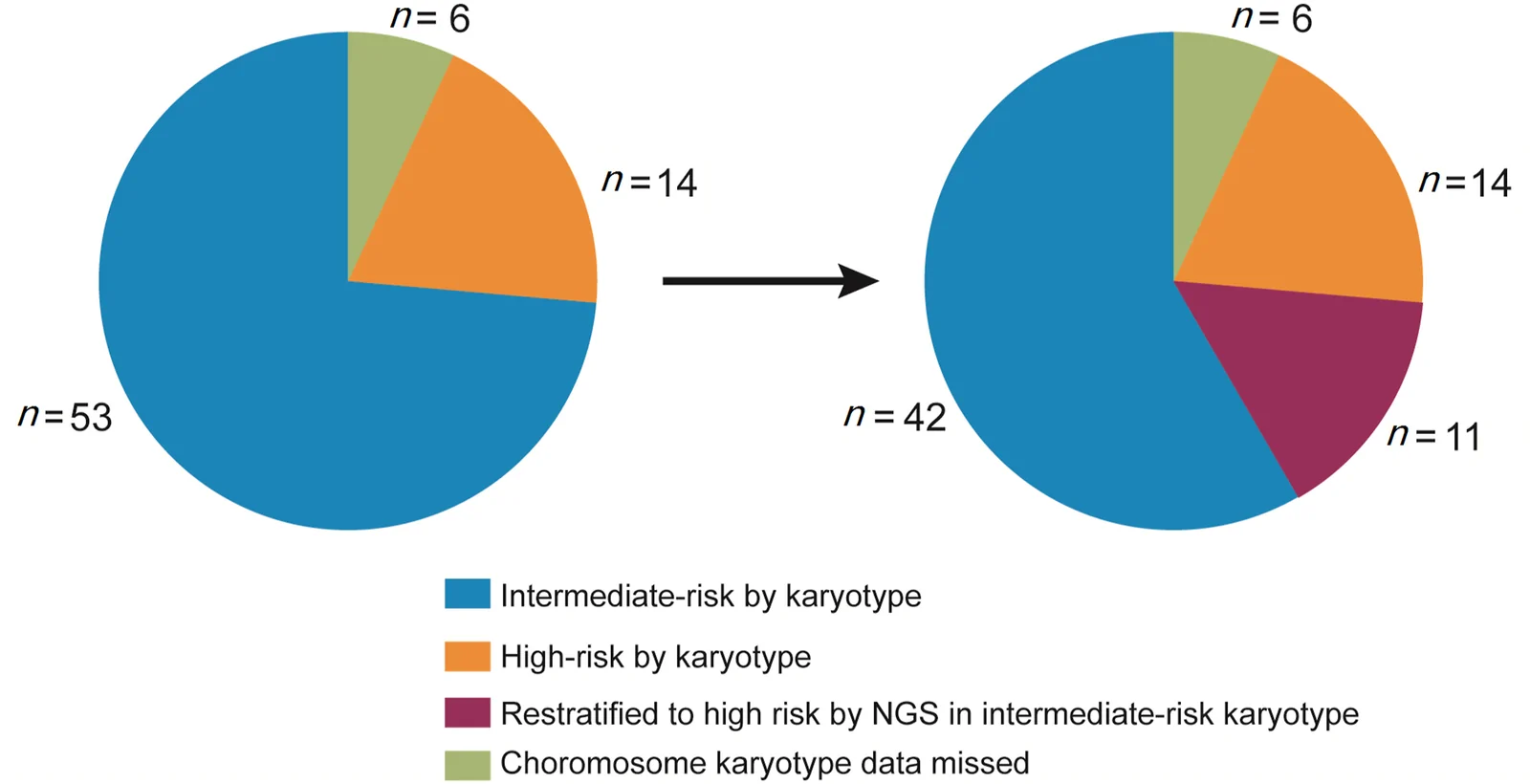
Fusion genes identified using NGS facilitate more precise stratification of the prognosis of patients with intermediate-risk karyotype acute myeloid leukemia. Of the 53 patients with medium-risk karyotypes, 11 (20.8%) demonstrated the presence of fusion genes with adverse prognostic implications via NGS, warranting their allocation to the high-risk category. The purple sector represents the newly discovered high-risk fusion genes from NGS, which allows patients with intermediate-risk karyotypes to be re-stratified as high-risk. NGS: Next-generation sequencing.

Among the 53 patients with intermediate-risk karyotypes, NGS identified fusion genes with well-established adverse prognostic significance in 11 of them. These abnormalities included five instances of *NUP98-NSD1* and five of *KMT2A* fusion (excluding *KMT2A-MLLT3*). Additionally, one patient who experienced a relapse in this study harbored a *PICALM-MLLT10* fusion, which has been previously associated with poor prognosis. The supplementary information obtained from NGS-detected fusions facilitated the re-stratification of 11 patients (20.8%) with an intermediate-risk karyotype into the high-risk category [Figure 4].

Among the five patients that were *NUP98-NSD1* gene-positive, four exhibited primary resistance to treatment with poor outcomes, whereas the remaining patient achieved long-term survival after undergoing hematopoietic stem cell transplantation during remission. Among the five patients with *KMT2A* fusion, two demonstrated primary drug resistance, two experienced disease relapse during treatment, and one attained long-term survival following transplantation during remission.

## Discussion

Clinically, a subset of patients with AML lacks high-risk genetic factors but still experiences drug resistance or relapse, highlighting the heterogeneity within this population. To investigate the potential causes of this heterogeneity, we performed NGS analysis of AML cases that were negative for fusion genes using RT-PCR at our center. This study specifically included a relatively high proportion of refractory or relapsed patients to enhance our understanding of patients with AML who demonstrate a poor prognosis despite the absence of known prognostic markers. Our findings revealed that approximately 30.0% of the enrolled patients with AML harbored fusion genes, with an average expression of 1.5 fusion transcripts per patient. These results highlight the remarkably high rate of missed detection of these genetic abnormalities. Furthermore, we observed varying frequencies of fusion genes among different disease states, with refractory patients exhibiting the highest proportion (42.3%), followed by relapsed and newly diagnosed patients. Importantly, refractory cases exhibit an alarming rate of undetected fusion genes, highlighting the clinical significance of the identified fusion events.

The most prevalent fusion genes observed in refractory patients were *KMT2A* and *NUP98* rearrangements. Furthermore, concurrent alterations in *EIF4A1* have been identified, necessitating further investigation of its pathogenesis. In the refractory cohort, novel occurrences of *EIF4A1-ACTB*, *LMO1-RIC3*, *EIF4A1-CIC*, *B4GALT7-NUP98*, and *KMT2A-CTNND1* fusion transcripts were identified for the first time. *KMT2A* rearrangements and *NUP98* gene changes have also been detected in patients with relapse. Relapsed cases exhibited a higher degree of fusion gene diversity than did refractory cases. Three previously unreported fusion transcripts, *BRE-MECOM*, *CDK6-HOXA11-AS1*, and *SEPT6-NKRF,* were identified for the first time in patients with relapsed AML. Notably, 6/10 novel transcripts (e.g., *KMT2A-CTNND1*) involved genes with established roles in leukemogenesis (*KMT2A* and *NUP98*) and chromatin regulation (*CTNND1*). Although their low variant allele frequency (VAF) (0.84%–8.64%) may suggest subcloned origins or transcriptional noise, recurrent detection in refractory cases (e.g., *EIF4A1-*rearrangements in two patients) warrants functional studies. For example, *CTNND1* (δ-catenin) is a Wnt/β-catenin activator, and *KMT2A* fusions may dysregulate *HOX* gene mechanisms, consistent with known AML pathways.

The refractory and relapsed patients exhibited a significant enrichment of fusion genes involving *KMT2A* and *NUP98,* along with various partner genes, similar to the findings of previous studies.^15^ In our samples, four patients harboring *KMT2A-MLLT3/MLLT10* were negative in initial RT-PCR but positive by NGS. Several technical and biological factors may explain these findings. Our multiplex RT-PCR panel covered breakpoints in more than 90.0% of the reported *KMT2A* fusions (according to the European Molecular Genetics Quality Network guidelines); however, atypical junctions or low-abundance transcripts may evade detection. The unbiased methodology and enhanced sequencing depth of NGS mitigate these limitations. The application of NGS enables the detection of atypical fusions and unconventional breakpoints within typical fusion genes, thereby providing comprehensive information into fusion gene profiles.^16^ While NGS identified fusions missed by RT-PCR, some may represent passenger events. However, further functional studies are required to confirm their leukemogenic roles. A total of 145 different forms of *KMT2A* gene fusions have been reported.^17^ *KMT2A* rearrangement occurs in 15.0% of adult patients with AML and is generally associated with poor prognosis, except for the *KMT2A-MLLT3* fusion. Seven specific *KMT2A* fusion genes account for 83.0%–90.0% of all fusion forms: *KMT2A-MLLT3*, *KMT2A-MLLT10*, *KMT2A-ELL*, *KMT2A-PTD*, *KMT2A-AF6*, *KMT2A-ENL*, and *KMT2A-SEPT6*.^18^ In this study, seven patients with *KMT2A* rearrangement were identified, resulting in five distinct fusion forms. Notably, a novel fusion form, *KMT2A-CTNND1,* was discovered. Additionally, *CTNND1* is a target gene regulated by *IKZF1*, and its high expression may be associated with poor prognosis in B-cell acute lymphoblastic leukemia (B-ALL).^19^^,^^20^ However, its role in AML has not yet been investigated.

*NUP98* fusion genes typically arise from chromosomal translocations, although fragment insertions can also be a causative factor. To date, up to 28 *NUP98* fusion genes have been documented.^21^ The *NUP98* fusion was initially identified in a case of t(7; 11) (p15; p15) AML, with *HOXA9* serving as the translocation partner gene.^22^ In our study, the highest incidence was observed for the *NUP98-NSD1* fusion gene. *NUP98* is located on 11p15, whereas *NSD1* is located on 5q35. Consequently, the corresponding chromosome karyotype should be t(5; 11) (q35; p15.5). However, all five patients expressing *NUP98-NSD1* fusion genes in this study exhibited normal karyotypes because both genes were positioned at the ends of the chromosomes, which are often overlooked during karyotyping. Owing to the variability of partner genes with *NUP98* and challenges associated with accurately identifying chromosome karyotypes, the detection of *NUP98* fusion genes is prone to oversight. Notably, there is an association between *FLT3-ITD* and *NUP98-NSD1* fusions, as 15.0% of *FLT3-ITD* patients carry the latter mutation. *FLT3-ITD* was detected in 82.0% of patients with *NUP98-NSD1* fusions.^23^ Patients harboring both mutations exhibited a significantly unfavorable prognosis, with a complete remission rate of only 27.0% and a three-year overall survival rate limited to 31.0%.^24^ Additionally, this study identified a novel fusion form, *NUP98-GALNTL4*, which encodes galactosyltransferase-I, a product of *GALNTL4*, that has been associated with Ehlers–Danlos syndrome and Larsen syndrome on Reunion Island through genetic mutations.^25^ In cases of cutaneous melanoma, *GALNTL4* single nucleotide polymorphisms have been associated with mRNA expression levels in skin tissues, thereby influencing prognosis via glycosylation pathways.^26^

The fusion gene *PICALM-MLLT10* is formed through the translocation t(10; 11) (p12.13; q14.21). In routine karyotype analysis, it can be easily mistaken for another translocation gene *KMT2A-MLLT10* on chromosomes 10 and 11. Hence, more accurate FISH detection methods are recommended. *PICALM-MLLT10* has been observed in both acute lymphoblastic leukemia (ALL) and AML cases.^27^ An investigation involving 18 patients with *PICALM-MLLT1*0 AML revealed that this group demonstrated a higher incidence of extramedullary involvement, an increased occurrence of treatment-related AML, an elevated relapse rate, and an overall survival rate comparable to medium-risk and high-risk subtypes.^28^^,^^29^

Eukaryotic initiation factor 4A (EIF4A), the enzymatic core of the EIF4F complex that is essential for translation initiation, plays a pivotal role in the oncogenic reprogramming of protein synthesis. Fusion events of *EIF4A* have not been previously reported in patients with AML. However, concurrent amplification or duplication of *EIF4F* has been observed in various human cancers in The Cancer Genome Atlas data.^30^ Additionally, a study investigating the therapeutic potential of inhibiting *EIF4A* in AML revealed that primary AML cells exhibit significantly higher levels of *EIF4A1* transcripts than other types of cancer. Furthermore, the inhibition of *EIF4A* demonstrated antileukemic activity in AML.^31^ The functional implications and significance of the fusion events involving *EIF4A1-ACTB* and *EIF4A1-CIC* in refractory AML remain unclear and require further investigation. While targeted RNA-seq identifies putative fusion transcripts, their genomic origins (e.g., chromosomal rearrangements *vs*. trans-splicing) require confirmation by DNA-based assays such as whole-genome sequencing or breakpoint-specific PCR.

NGS not only provides more biological information but also enables the accurate prognostic stratification of patients, guiding treatment choices. In this study, 20.8% of patients with medium-risk karyotype AML carried fusion genes with poor prognostic significance. The detection of fusion genes using NGS can contribute to the precise risk stratification of patients with intermediate-risk AML. However, the prognostic significance of some new and rare fusion genes detected in these medium-risk karyotypes remains unclear (e.g., *LMO1-RIC3*, *PRDM16-SKI*, *BRE-MECOM*, *CDK6-HOXA11-AS1*, and *SEPT6-NKRF*). Despite the refractory reality or possibility of relapse, the low VAF value (0.84%–8.64%) and the unclear role they play in drug resistance and relapse pose challenges in establishing a foundation for further prognostic stratification. Consequently, there may be a potential underestimation of the proportion of high-risk fusion genes in cases with normal karyotypes. Three patients with *KMT2A* and *NUP98* fusions demonstrated prolonged survival, implying that enhancing intensive treatment for high-risk patients could potentially improve their prognosis.

Each fusion gene exhibits distinct biological functions that act as potent pathogenic factors. Fusion transcription generates oncoproteins that trigger leukemia; therefore, a dominant clone carrying a specific fusion gene can be detected in leukemic cells. However, based on the findings of a comprehensive sequencing analysis, researchers employed whole-exome (*n* = 150 cases) or whole-genome (*n* = 50 cases) sequencing, and RNA sequencing of 80 samples with fusion genes, and identified 118 fusion genes. The average number of fusion genes per sample was 1.5, ranging from 0 to 8. The functions of these fusion genes varied depending on the specific breakpoints within them. Certain fusion genes undergo transcription and translation to produce oncogenic fusion proteins. Conversely, out-of-frame fusion results in the inactivation of partner genes.^32^ In our study, we observed that of the 21 samples positive for fusion genes, the simultaneous expression of complementary fusion genes was detected in six samples, while an additional fusion gene was identified in four specimens. Co-expression of complementary fusion genes is a prevalent phenomenon in AML and is potentially present in approximately 50% of tumor cells harboring fusion genes. *TOP1-NUP98* was observed in treatment-related MDS without *NUP98-TOP1*, whereas both *NUP98-TOP1* and *TOP1-NUP98* were detected in patients with newly diagnosed AML. Hence, the emergence of *NUP98-TOP1* may be associated with disease progression.^33^ The mechanisms and functions of various fusion genes, including both complementary and non-complementary forms, warrant in-depth research. This cross-sectional study describes the spectrum of fusion transcripts in PCR-negative AML. While these findings highlight the potential of targeted NGS to reveal hidden molecular features, the causal relationships between specific fusions and treatment resistance require prospective validation through cohort studies. NGS can fulfill the requirements for the simultaneous detection of multiple fusion genes, thereby facilitating a more comprehensive understanding of AML pathogenesis. To address the causal relationship between NGS-detected fusions and clinical outcomes, a multicenter prospective study incorporating serial NGS monitoring and randomized treatment adjustments based on the fusion status is necessary.

This study was an exploratory analysis that used targeted NGS to identify fusion genes in adult patients with AML, particularly in refractory/relapsed cases. Notably, the clinical application of NGS for detecting fusion genes requires patient selectivity. NGS methods are highly effective for refractory or relapsed patients with poor prognostic indicators. The application of NGS can also be extended to address clinical needs in cases in which a potential fusion gene is not detected after thoroughly considering the presence of concurrent gene mutations. However, NGS cannot replace chromosomal karyotyping. In this study, we used retrospective samples from a single research center to analyze potential fusion genes in PCR-negative AML. However, this study has certain limitations. The sample size was limited and derived from a specific population diagnosed with AML between 2016 and 2017. During this period, information on gene mutations obtained through NGS was unavailable in some cases, thereby preventing a comprehensive analysis of molecular aberrations.

## Conclusion

In general, targeted NGS analysis revealed a substantial number of fusion genes in our retrospective AML samples, thereby elucidating the molecular biological mechanisms underlying the unfavorable prognosis observed in certain refractory and relapsed patients. Moreover, it facilitates a more precise risk stratification for medium-risk karyotype AML diagnosis. The detection of fusion genes using NGS significantly enhances our understanding of AML molecular genetics and provides an efficient tool for diagnosis and targeted therapy of AML. These results highlight the need for iterative hypothesis-driven research to bridge the gap between molecular discovery and clinical translation.

## Authors contribution

Conceptualization: Yu Li and Wei Guan; methodology: Yangliu Shao, Nan Wang, and Yan Li; formal analysis: Ketao Wang; investigation: Lei Zhou, Yi Ding, Maoquan Wang, Wei Zhou, Yu Jing, Yonghui Li, and Lili Wang; resources: Li Yu and Wei Guan; data curation: Wei Guan, Lei Zhou, and Yangliu Shao; writing- original draft preparation: Wei Guan and Ketao Wang; visualization: Wei Guan and Nan Wang; supervision: Li Yu and Daihong Liu; project administration: Li Yu and Wei Guan. All the authors have read and approved the final paper.

## Ethics statement

This study was conducted in accordance with the *Declaration of Helsinki* (2013 revision). Informed consent has been obtained from all patients. All sampling procedures were approved by the Institutional Review Committee of the PLA general hospital (No. S2016-076-01). The privacy rights of human subjects must always be observed.

## Data availability statement

The datasets used in the current study are available from the corresponding author on reasonable request.

## Declaration of generative AI and AI-assisted technologies in the writing process

The authors declare that generative artificial intelligence (AI) and AI assisted technologies were not used in the writing process or any other process during the preparation of this manuscript.

## Funding

This work was supported by the 10.13039/100014717National Natural Science Foundation of China (No.82100164, 82302692), the 10.13039/501100012425Capital Medical University Research Cultivation Fund (No. PYZ22099), and the Guangdong Provincial Medical Science and Technology Research Fund Project (No. A2024190).

## Conflict of interest

The authors declare that they have no known competing financial interests or personal relationships that could have appeared to influence the work reported in this paper.
