# Supplementary material for: Insights into the biological features and improved diagnostics of adult acute myeloid leukemia via fusion genes identified through targeted next-generation sequencing
Source: Cancer Pathog Ther. 2025 Jun 10;4(1):64–71. doi: 10.1016/j.cpt.2025.06.003 (PMC12723017; doi:10.1016/j.cpt.2025.06.003)
Supplement: Multimedia component 1 [file mmc1.docx]

**Supplementary Table 1:** **General characteristics of the positive controls.**

| **Characteristics** | **Value** |
| --- | --- |
| **Age (years)** | 34 (12–64) |
| **Male** | 13 (76.5) |
| **Disease** |  |
| **Acute myeloid leukemia** | 11 (64.7) |
| **Lymphoma** | 3 (17.6) |
| **Chronic myeloid leukemia** | 2 (11.8) |
| **Multiple myeloma** | 1 (5.9) |
| **WBC count at diagnosis (×10^9^/L)** | 39.5 (1.28–158.31) |
| **Hemoglobin count at diagnosis (g/L)** | 82.3 (55–111) |
| **Platelet count at diagnosis (×10^9^/L)** | 46.9 (25–109) |
| **Blasts in sample (%)** | 44 (2–80.4) |

Data are presented as *n* (%) or median (range). AML: Acute myeloid leukemia; CML: Chronic myeloid leukemia; MM: Multiple myeloma; WBC: White blood cells.

**Supplementary Table 2:** **List of AML-associated fusion genes detected by multiplex nested RT-PCR.**

| **Name of fusion gene** |
| --- |
| *RUNX1-EVI1* |
| *RUNX1-MTG16* |
| *RUNX1-RUNX1T1* |
| *BCR-ABL* |
| *FIP1LT-PDGFR* |
| *TEL-PDGFR* |
| *TEL-ABL1* |
| *KMT2A-AF9* |
| *KMT2A-AF6* |
| *KMT2A-AF10* |
| *PML-RARA* |
| *NPM1-RARA* |
| *TLS-ERG* |
| *CBFβ-MYH11* |
| *DEK-NUP214* |
| *SET-NUP214* |
| *NUP98-HOXA9* |
| *KMT2A-PTD* |
| *KMT2A-ELL* |
| *KMT2A-ENL* |

AML: Acute myeloid leukemia; *BCR*-*ABL*: Breakpoint cluster region - abelson tyrosine kinase; *CBFβ*-*MYH11*: Core binding factor beta-myosin heavy chain 11; *DEK*-*NUP214*: DEK-nucleoporin 214; *FIP1LT*-*PDGFR*: FIP1 like 1-platelet derived growth factor receptor; *KMT2A*-*AF10*: Lysine methyltransferase 2A-mixed-lineage leukemia translocated to 10; *KMT2A*-*AF6*: Lysine methyltransferase 2A-afadin; *KMT2A*-*AF9*: Lysine methyltransferase 2A-mixed-lineage leukemia translocated to 3 ; *KMT2A*-*ELL*: Lysine methyltransferase 2A-elongation factor for RNA polymerase II ; *KMT2A*-*ENL*: Lysine methyltransferase 2A-mixed-lineage leukemia translocated to 1 ; *KMT2A*-*PTD*: Lysine methyltransferase 2A partial tandem duplication; *NPM1*-*RARA*: Nucleophosmin 1- retinoic acid receptor alpha; *NUP98*-*HOXA9*: Nucleoporin 98-homeobox A9; *PML*-*RARA*: Promyelocytic leukemia-retinoic acid receptor alpha; RT-PCR: Reverse transcription-polymerase chain reaction; *RUNX1*-*EVI1*: RUNX family transcription factor 1-ecotropic viral integration site 1; *RUNX1*-*MTG16*: RUNX family transcription factor 1-MTG16 fusion; *RUNX1*-*RUNX1T1*: RUNX family transcription factor 1-RUNX1 partner transcript 1; *SET*-*NUP214*: SET-nucleoporin 214; *TEL*-*ABL1*: ETS Variant transcription factor 6-abelson murine leukemia viral oncogene homolog 1 ; *TEL*-*PDGFR*: ETV6-platelet derived growth factor receptor; *TLS*-*ERG*: Fused in sarcoma-ETS-related gene.

**Supplementary Table 3:** **Gene panel list by next-generation sequencing.**

| **Gene name** | **Other gene names** | **Gene ID** | **Location** | **Number of fusions,*n*** |
| --- | --- | --- | --- | --- |
| *KMT2A* | *ALL-1/CXXC7/HRX/HTRX1/MLL/MLL-AF9/MLL/GAS7/MLL1/MLL1A/TET1-MLL/TRX1/WDSTS* | 4297 | 11q23 | 66 |
| *ETV6* | *TEL/TEL/ABL/THC5* | 2120 | 12p13 | 41 |
| *ALK* | *CD246/NBLST3* | 238 | 2p23 | 35 |
| *RARA* | *NR1B1/RAR* | 5914 | 17q21 | 34 |
| *RUNX1* | *AML1/AML1-EVI-1/AMLCR1/CBF2alpha/CBFA2/ EVI-1/PEBP2aB/PEBP2alpha* | 861 | 21q22.3 | 34 |
| *NUP98* | *ADIR2/NUP196/NUP96* | 4928 | 11p15.5 | 33 |
| *BCL6* | *BCL5/BCL6A/LAZ3/ZBTB27/ZNF51* | 604 | 3q27 | 31 |
| *PDGFRB* | *CD140B/IBGC4/IMF1/JTK12/KOGS/PDGFR/ PDGFR-1/PDGFR1/PENTT* | 5159 | 5q33.1 | 28 |
| *EWSR1* | *EWS/EWS-FLI1/bK984G1.4* | 2130 | 22q12.2 | 27 |
| *FOXP1* | *12CC4/HSPC215/MFH/QRF1/hFKH1B* | 27086 | 3p14.1 | 27 |
| *FGFR1* | *BFGFR/CD331/CEK/FGFBR/FGFR-1/FLG/FLT-2/ FLT2/HBGFR/HH2/HRTFDS/KAL2/N-SAM/ OGD/bFGF-R-1* | 2260 | 8p11.23-p11.22 | 22 |
| *ROS1* | *MCF3/ROS/c-ros-1* | 6098 | 6q22 | 22 |
| *PAX5* | *ALL3/BSAP* | 5079 | 9p13 | 22 |
| *RET* | *CDHF12/CDHR16/HSCR1/MEN2A/MEN2B/ MTC1/PTC/RET-ELE1/RET51* | 5979 | 10q11.2 | 21 |
| *JAK2* | *JTK10/THCYT3* | 3717 | 9p24 | 18 |
| *MYC* | *MRTL/MYCC/bHLHe39/c-Myc* | 4609 | 8q24.21 | 17 |
| *HMGA2* | *BABL/HMGI-C/HMGIC/LIPO/STQTL9* | 8091 | 12q15 | 17 |
| *ABL1* | *ABL/JTK7/bcr/abl/c-ABL/c-ABL1/p150/v-abl* | 25 | 9q34.1 | 16 |
| *LPP* | *-* | 4026 | 3q28 | 16 |
| *ERG* | *erg-3/p55* | 2078 | 21q22.3 | 14 |
| *KAT6A* | *MOZ/MRD32/MYST-3/MYST3/RUNXBP2/ ZC2HC6A/ZNF220* | 7994 | 8p11 | 14 |
| *MLLT10* | *AF10* | 8028 | 10p12 | 13 |
| *ETV1* | *ER81* | 2115 | 7p21.3 | 13 |
| *FUS* | *ALS6/ETM4/FUS1/HNRNPP2/POMP75/TLS* | 2521 | 16p11.2 | 12 |
| *PBX1* | *-* | 5087 | 1q23 | 11 |
| *MYB* | *Cmyb/c-myb/c-myb_CDS/efg* | 4602 | 6q22-q23 | 11 |
| *PLAG1* | *PSA/SGPA/ZNF912* | 5324 | 8q12 | 11 |
| *ETV4* | *E1A-F/E1AF/PEA3/PEAS3* | 2118 | 17q21.31 | 11 |
| *BCR* | *ALL/BCR1/CML/D22S11/D22S662/PHL* | 613 | 22q11.23 | 10 |
| *MECOM* | *AML1-EVI-1/EVI1/MDS1/MDS1-EVI1/PRDM3* | 2122 | 3q26.2 | 10 |
| *NUP214* | *CAIN/CAN/D9S46E/N214/p250* | 8021 | 9q34.1 | 10 |
| *TFE3* | *RCCP2/RCCX1/TFEA/bHLHe33* | 7030 | Xp11.22 | 10 |
| *CREBBP* | *CBP/KAT3A/RSTS* | 1387 | 16p13.3 | 10 |
| *CCDC6* | *D10S170/H4/PTC/TPC/TST1* | 8030 | 10q21 | 10 |
| *CLTC* | *CHC/CHC17/CLH-17/CLTCL2/Hc* | 1213 | 17q23.1 | 10 |
| *COL1A1* | *EDSC/OI1/OI2/OI3/OI4* | 1277 | 17q21.33 | 9 |
| *ACTB* | *BRWS1/PS1TP5BP1* | 60 | 7p22 | 9 |
| *BCL2* | *Bcl-2/PPP1R50* | 596 | 18q21.3 | 8 |
| *FGFR3* | *ACH/CD333/CEK2/HSFGFR3EX/JTK4* | 2261 | 4p16.3 | 8 |
| *TCF3* | *E2A/E47/ITF1/TCF-3/VDIR/bHLHb21* | 6929 | 19p13.3 | 8 |
| *MAML2* | *MAM-3/MAM2/MAM3/MLL-MAML2* | 84441 | 11q21 | 8 |
| *TMPRSS2* | *PP9284/PRSS10* | 7113 | 21q22.3 | 8 |
| *AFF3* | *LAF4/MLLT2-like* | 3899 | 2q11.2-q12 | 8 |
| *CCND1* | *BCL1/D11S287E/PRAD1/U21B31* | 595 | 11q13 | 7 |
| *JAZF1* | *TIP27/ZNF802* | 221895 | 7p15.2-p15.1 | 7 |
| *CCND2* | *KIAK0002/MPPH3* | 894 | 12p13 | 7 |
| *BCL11B* | *ATL1/ATL1-alpha/ATL1-beta/ATL1-delta/ATL1-gamma/CTIP-2/CTIP2/RIT1/ZNF856B/hRIT1-alpha* | 64919 | 14q32.2 | 7 |
| *TPM3* | *CAPM1/CFTD/HEL-189/HEL-S-82p/NEM1/OK/SW-cl.5/TM-5/TM3/TM30/TM30nm/TM5/TPMsk3/ TRK/hscp30* | 7170 | 1q21.2 | 7 |
| *NPM1* | *B23/NPM* | 4869 | 5q35.1 | 6 |
| *PDGFRA* | *CD140A/PDGFR-2/PDGFR2/RHEPDGFRA* | 5156 | 4q12 | 6 |
| *PICALM* | *CALM/CLTH/LAP* | 8301 | 11q14 | 6 |
| *SS18* | *SSXT/SYT* | 6760 | 18q11.2 | 6 |
| *TAL1* | *SCL/TCL5/bHLHa17/tal-1* | 6886 | 1p32 | 6 |
| *AKT3* | *MPPH/MPPH2/PKB-GAMMA/PKBG/PRKBG/ RAC-PK-gamma/RAC-gamma/STK-2* | 10000 | 1q44 | 6 |
| *USP6* | *HRP1/TRE17/TRE2/TRESMCR/Tre-2/USP6-short* | 9098 | 17p13 | 6 |
| *TFG* | *HMSNP/SPG57/TF6/TRKT3* | 10342 | 3q12.2 | 6 |
| *SLC45A3* | *IPCA-2/IPCA-6/IPCA-8/IPCA6/PCANAP2/PCANAP6/PCANAP8/PRST* | 85414 | 1q32.1 | 6 |
| *TAF15* | *Npl3/RBP56/TAF2N/TAFII68* | 8148 | 17q11.1-q11.2 | 5 |
| *PAX3* | *CDHS/HUP2/WS1/WS3* | 5077 | 2q35 | 5 |
| *TRIM24* | *PTC6/RNF82/TF1A/TIF1/TIF1A/TIF1ALPHA/ hTIF1* | 8805 | 7q32-q34 | 5 |
| *CDK6* | *MCPH12/PLSTIRE* | 1021 | 7q21-q22 | 5 |
| *PRDM16* | *CMD1LL/LVNC8/MEL1/PFM13* | 63976 | 1p36.23-p33 | 5 |
| *CBFA2T3* | *ETO2/MTG16/MTGR2/ZMYND4* | 863 | 16q24 | 4 |
| *PRKAR1A* | *ACRDYS1/ADOHR/CAR/CNC/CNC1/PKR1/ PPNAD1/PRKAR1/TSE1* | 5573 | 17q24.2 | 4 |
| *TLX3* | *HOX11L2/RNX* | 30012 | 5q35.1 | 4 |
| *BCL3* | *BCL4/D19S37* | 602 | 19q13.1-q13.2 | 4 |
| *DDIT3* | *CEBPZ/CHOP/CHOP-10/CHOP10/GADD153* | 1649 | 12q13.1-q13.2 | 3 |
| *FOXO4* | *AFX/AFX1/MLLT7* | 4303 | Xq13.1 | 3 |
| *HOXA9* | *ABD-B/HOX1/HOX1.7/HOX1G* | 3205 | 7p15.2 | 3 |
| *MALT1* | *IMD12/MLT/MLT1* | 10892 | 18q21 | 3 |
| *NKX2-5* | *CHNG5/CSX/CSX1/HLHS2/NKX2.5/NKX2E/*  *NKX4-1/VSD3* | 1482 | 5q34 | 3 |
| *PML* | *MYL/PP8675/RNF71/TRIM19* | 5371 | 15q22 | 3 |
| *RPN1* | *OST1/RBPH1* | 6184 | 3q21.3 | 3 |
| *CCND3* | *-* | 896 | 6p21 | 3 |
| *RHOH* | *ARHH/TTF* | 399 | 4p13 | 3 |
| *FOXO1* | *FKH1/FKHR/FOXO1A* | 2308 | 13q14.1 | 3 |
| *NTRK1* | *MTC/TRK/TRK1/TRKA/Trk-A/p140-TrkA* | 4914 | 1q21-q22 | 3 |
| *TBL1XR1* | *C21/DC42/IRA1/TBLR1* | 79718 | 3q26.32 | 3 |
| *EBF1* | *COE1/EBF/O/E-1/OLF1* | 1879 | 5q34 | 3 |
| *AFF1* | *AF4/MLLT2/PBM1* | 4299 | 4q21 | 2 |
| *BIRC3* | *AIP1/API2/CIAP2/HAIP1/HIAP1/MALT2/MIHC/ RNF49/c-IAP2* | 330 | 11q22 | 2 |
| *CBFB* | *PEBP2B* | 865 | 16q22.1 | 2 |
| *FIP1L1* | *FIP1/Rhe/hFip1* | 81608 | 4q12 | 2 |
| *HFE2* | *HFE2A/HJV/JH/RGMC* | 148738 | 1q21.1 | 2 |
| *HIP1* | *HIP-I/ILWEQ/SHON/SHONbeta/SHONgamma* | 3092 | 7q11.23 | 2 |
| *HOXA11* | *HOX1/HOX1I* | 3207 | 7p15.2 | 2 |
| *MAF* | *AYGRP/CCA4/CTRCT21/c-MAF* | 4094 | 16q22-q23 | 2 |
| *MYO18A* | *MYSPDZ/SPR210* | 399687 | 17q11.2 | 2 |
| *SET* | *2PP2A/I2PP2A/IGAAD/IPP2A2/PHAPII/TAF-I/ TAF-IBETA* | 6418 | 9q34 | 2 |
| *TLX1* | *HOX11/TCL3* | 3195 | 10q24 | 2 |
| *CRLF2* | *CRL2/CRLF2Y/TSLPR* | 64109 | Xp22.3; Yp11.3 | 2 |
| *BCL9* | *LGS* | 607 | 1q21 | 2 |
| *LMO1* | *RBTN1/RHOM1/TTG1* | 4004 | 11p15 | 2 |
| *IRF4* | *LSIRF/MUM1/NF-EM5/SHEP8* | 3662 | 6p25.3 | 2 |
| *BCL10* | *CARMEN/CIPER/CLAP/IMD37/c-E10/mE10* | 8915 | 1p22 | 2 |
| *RANBP17* | *-* | 64901 | 5q34 | 2 |
| *KDM4C* | *GASC1/JHDM3C/JMJD2C/TDRD14C* | 23081 | 9p24.1 | 2 |
| *PDCD1LG2* | *B7DC/Btdc/CD273/PD-L2/PDCD1L2/PDL2/ bA574F11.2* | 80380 | 9p24.2 | 2 |
| *RABEP1* | *RAB5EP/RABPT5* | 9135 | 17p13.2 | 2 |
| *CIC* | *-* | 23152 | 19q13.2 | 2 |
| *NOTCH1* | *AOS5/AOVD1/TAN1/hN1* | 4851 | 9q34.3 | 2 |
| *ERVW-1* | *ENV/ENVW/ERVWE1/HERV-7q/HERV-W-ENV/ HERV7Q/HERVW/HERVWENV* | 30816 | 7q21.2 | 2 |
| *SSX1* | *CT5.1/SSRC* | 6756 | Xp11.23 | 2 |
| *A2M* | *A2MD/CPAMD5/FWP007/S863-7* | 2 | 12p13.31 | 1 |
| *ABI1* | *ABI-1/ABLBP4/E3B1/NAP1BP/SSH3BP/SSH3BP1* | 10006 | 10p11.2 | 1 |
| *ABL2* | *ABLL/ARG* | 27 | 1q25.2 | 1 |
| *ACTN4* | *ACTININ-4/FSGS/FSGS1* | 81 | 19q13 | 1 |
| *BCOR* | *ANOP2/MAA2/MCOPS2* | 54880 | Xp11.4 | 1 |
| *BIN2* | *BRAP-1* | 51411 | 12q13 | 1 |
| *CBFA2T2* | *EHT/MTGR1/ZMYND3/p85* | 9139 | 20q11 | 1 |
| *DDX10* | *HRH-J8* | 1662 | 11q22-q23 | 1 |
| *DEK* | *D6S231E* | 7913 | 6p22.3 | 1 |
| *ELL* | *C19orf17/ELL1/MEN/PPP1R68* | 8178 | 19p13.1 | 1 |
| *EPS15* | *AF-1P/AF1P/MLLT5* | 2060 | 1p32 | 1 |
| *FOXO3* | *AF6q21/FKHRL1/FKHRL1P2/FOXO2/FOXO3A* | 2309 | 6q21 | 1 |
| *GIT2* | *CAT-2/CAT2* | 9815 | 12q24.1 | 1 |
| *HLF* | *-* | 3131 | 17q22 | 1 |
| *HOXA13* | *HOX1/HOX1J* | 3209 | 7p15.2 | 1 |
| *HOXC11* | *HOX3H* | 3227 | 12q13.3 | 1 |
| *HOXD13* | *BDE/BDSD/HOX4I/SPD* | 3239 | 2q31.1 | 1 |
| *IL3* | *IL-3/MCGF/MULTI-CSF* | 3562 | 5q31.1 | 1 |
| *MAFB* | *KRML/MCTO* | 9935 | 20q12 | 1 |
| *MKL1* | *BSAC/MAL/MRTF-A* | 57591 | 22q13 | 1 |
| *MLF1* | *-* | 4291 | 3q25.1 | 1 |
| *MLLT1* | *ENL/LTG19/YEATS1* | 4298 | 19p13.3 | 1 |
| *MLLT11* | *AF1Q* | 10962 | 1q21 | 1 |
| *MLLT3* | *AF9/YEATS3* | 4300 | 9p22 | 1 |
| *MLLT4* | *AF6/MLL-AF6* | 4301 | 6q27 | 1 |
| *MLLT6* | *AF17* | 4302 | 17q21 | 1 |
| *MYH11* | *AAT4/FAA4/SMHC/SMMHC* | 4629 | 16p13.11 | 1 |
| *NUMA1* | *NMP-22/NUMA* | 4926 | 11q13 | 1 |
| *PDGFB* | *IBGC5/PDGF-2/PDGF2/SIS/SSV/c-sis* | 5155 | 22q13.1 | 1 |
| *PRRX1* | *AGOTC/PHOX1/PMX1/PRX-1/PRX1* | 5396 | 1q24 | 1 |
| *RBM15* | *OTT/OTT1/SPEN* | 64783 | 1p13 | 1 |
| *RUNX1T1* | *AML1-MTG8/AML1T1/CBFA2T1/CDR/ETO/MTG8/ ZMYND2* | 862 | 8q22 | 1 |
| *6-Sep* | *SEP2/SEPT2* | 23157 | Xq24 | 1 |
| *SSX2* | *CT5.2/CT5.2A/HD21/HOM-MEL-40/SSX* | 6757 | Xp11.22 | 1 |
| *STAT5B* | *STAT5* | 6777 | 17q11.2 | 1 |
| *STIL* | *MCPH7/SIL* | 6491 | 1p32 | 1 |
| *ZBTB16* | *PLZF/ZNF145* | 7704 | 11q23.1 | 1 |
| *ZMYM2* | *FIM/MYM/RAMP/SCLL/ZNF198* | 7750 | 13q11-q12 | 1 |
| *MYCN* | *MODED/N-myc/NMYC/ODED/bHLHe37* | 4613 | 2p24.3 | 1 |
| *TERT* | *CMM9/DKCA2/DKCB4/EST2/PFBMFT1/TCS1/TP2/TRT/hEST2/hTRT* | 7015 | 5p15.33 | 1 |
| *NFKB2* | *CVID10/H2TF1/LYT-10/LYT10/NF-kB2/p100/p52* | 4791 | 10q24 | 1 |
| *CEBPG* | *GPE1BP/IG/EBP-1* | 1054 | 19q13.11 | 1 |
| *LHX2* | *LH2/hLhx2* | 9355 | 9q33.3 | 1 |
| *TPD52* | *D52/N8L/PC-1/PrLZ/hD52* | 7163 | 8q21.13 | 1 |
| *ABCA13* | *-* | 154664 | 7p12.3 | 1 |
| *LCK* | *IMD22/LSK/YT16/p56lck/pp58lck* | 3932 | 1p34.3 | 1 |
| *GPR34* | *LYPSR1* | 2857 | Xp11.4 | 1 |
| *IRF8* | *H-ICSBP/ICSBP/ICSBP1/IMD32A/IMD32B/IRF-8* | 3394 | 16q24.1 | 1 |
| *BANK1* | *BANK* | 55024 | 4q24 | 1 |
| *TNFSF13* | *APRIL/CD256/TALL-2/TALL2/TRDL-1/UNQ383/ PRO715/ZTNF2* | 8741 | 17p13.1 | 1 |
| *IRS4* | *IRS-4/PY160* | 8471 | Xq22.3 | 1 |
| *DDX6* | *HLR2/P54/RCK* | 1656 | 11q23.3 | 1 |
| *CEBPA* | *C/EBP-alpha/CEBP* | 1050 | 19q13.1 | 1 |
| *GRIP1* | *GRIP* | 23426 | 12q14.3 | 1 |
| *LMO2* | *RBTN2/RBTNL1/RHOM2/TTG2* | 4005 | 11p13 | 1 |
| *LYL1* | *bHLHa18* | 4066 | 19p13.2 | 1 |
| *PPP1CB* | *HEL-S-80p/PP-1B/PP1B/PP1beta/PPP1CD* | 5500 | 2p23 | 1 |
| *EGFR* | *ERBB/ERBB1/HER1/NISBD2/PIG61/mENA* | 1956 | 7p12 | 1 |
| *BMI1* | *FLVI2/BMI1/PCGF4/RNF51/flvi-2/bmi-1* | 648 | 10p11.23 | 1 |
| *FCRL4* | *CD307d/FCRH4/IGFP2/IRTA1* | 83417 | 1q21 | 1 |
| *BACH2* | *BTBD25* | 60468 | 6q15 | 1 |
| *KDSR* | *DHSR/FVT1/SDR35C1* | 2531 | 18q21.3 | 1 |
| *IGF2BP1* | *CRD-BP/CRDBP/IMP-1/IMP1/VICKZ1/ZBP1* | 10642 | 17q21.32 | 1 |
| *DUSP22* | *JKAP/JSP-1/JSP1/LMW-DSP2/LMWDSP2/*  *MKP-x/MKPX/VHX* | 56940 | 6p25.3 | 1 |
| *BCL11A* | *BCL11A-L/BCL11A-S/BCL11A-XL/BCL11a-M/ CTIP1/EVI9/HBFQTL5/ZNF856* | 53335 | 2p16.1 | 1 |
| *EPOR* | *EPO-R* | 2057 | 19p13.3-p13.2 | 1 |
| *CD44* | *CDW44/CSPG8/ECMR-III/HCELL/HUTCH-I/IN/ LHR/MC56/MDU2/MDU3/MIC4/Pgp1* | 960 | 11p13 | 1 |
| *PAFAH1B2* | *HEL-S-303* | 5049 | 11q23 | 1 |
| *TCF12* | *CRS3/HEB/HTF4/HsT17266/TCF-12/bHLHb20* | 6938 | 15q21 | 1 |
| *REL* | *C-Rel* | 5966 | 2p13-p12 | 1 |
| *EPC1* | *Epl1* | 80314 | 10p11 | 1 |
| *CNN3* | *-* | 1266 | 1p22-p21 | 1 |
| *TAL2* | *-* | 6887 | 9q32 | 1 |
| *PCSK7* | *LPC/PC7/PC8/SPC7* | 9159 | 11q23-q24 | 1 |
| *MAST2* | *MAST205/MTSSK* | 23139 | 1p34.1 | 1 |
| *MUC1* | *ADMCKD/ADMCKD1/CA 15-3/CD227/EMA/ H23AG/KL-6/MAM6/MCD/MCKD/MCKD1/MUC-1/MUC-1/SEC/MUC-1/X/MUC1/ZD/PEM/PEMT/ PUM* | 4582 | 1q21 | 1 |
| *CEBPB* | *C/EBP-beta/IL6DBP/NF-IL6/TCF5* | 1051 | 20q13.1 | 1 |
| *CHST11* | *C4ST/C4ST-1/C4ST1/HSA269537* | 50515 | 12q | 1 |
| *CEBPD* | *C/EBP-delta/CELF/CRP3/NF-IL6-beta* | 1052 | 8p11.2-p11.1 | 1 |
| *WHSC1* | *MMSET/NSD2/REIIBP/TRX5/WHS* | 7468 | 4p16.3 | 1 |
| *ZC3H12D* | *C6orf95/MCPIP4/TFL/dJ281H8.1/p34* | 340152 | 6q25.1 | 1 |
| *FCGR2B* | *CD32/CD32B/FCG2/FCGR2/IGFR2* | 2213 | 1q23 | 1 |
| *HOXA10* | *HOX1/HOX1.8/HOX1H/PL* | 3206 | 7p15.2 | 1 |
| *CEBPE* | *C/EBP-epsilon/CRP1* | 1053 | 14q11.2 | 1 |
| *ID4* | *IDB4/bHLHb27* | 3400 | 6p22.3 | 1 |
| *SPIB* | *SPI-B* | 6689 | 19q13.3-q13.4 | 1 |
| *TENM2* | *ODZ2/TEN-M2/TNM2/ten-2* | 57451 | 5q34 | 1 |
| *LHX4* | *CPHD4* | 89884 | 1q25.2 | 1 |
| *CCNE1* | *CCNE/pCCNE1* | 898 | 19q12 | 1 |

*A2M:* Alpha-2-macroglobulin*; ABI1:* Abl interactor 1*; ABL1:* ABL proto-oncogene 1, non-receptor tyrosine kinase*; ABL2:* ABL proto-oncogene 2, non-receptor tyrosine kinase*; ACTB:* Actin beta*; ACTN4:* Actinin alpha 4*; AFF3:* AF4/FMR2 family member 3; *AKT3:* AKT serine/threonine kinase 3;*ALK:* Anaplastic lymphoma kinase; *BACH2:* BTB domain and CNC homolog 2; *BCL10:* B-cell lymphoma/leukemia 10; *BCL11A:* B-cell lymphoma/leukemia 11A; *BCL11B:* B-cell lymphoma/leukemia 11B; *BCL2:* B-cell lymphoma 2; *BCL3:* B-cell lymphoma 3; *BCL6:* B-cell lymphoma 6; *BCL9:* B-cell lymphoma 9; *BMI1:* B lymphoma Mo-MLV insertion region 1 homolog; *BANK1:* B-cell scaffold protein with ankyrin repeats 1; *BCR:* BCR activator of RhoGEF and GTPase; *CBFA2T3:* Core-binding factor subunit beta 2, translocated to 3; *CBFA2T2:* Core-binding factor subunit beta 2, translocated to 2*; CD44:* Cluster differentiation 44; *CDK6:* Cyclin-dependent kinase 6; *CNN3:* Calponin 3; *COL1A1:* Collagen type I alpha 1 chain; *CREBBP:* CREB binding protein; *DEK:* DEK proto-oncogene; *DDIT3:* DNA damage inducible transcript 3; *DDX10:* DEAD-box helicase 10; *DDX6:* DEAD-box helicase 6; *DUSP22:* Dual specificity phosphatase 22; *EGFR:* Epidermal growth factor receptor; *EPC1:* Enhancer of polycomb homolog 1; *EPOR:* Erythropoietin receptor; *ETV1:* ETS variant transcription factor 1; *ETV4:* ETS variant transcription factor 4*; ETV6:* ETS variant transcription factor 6; *EPS15:* Epidermal growth factor receptor pathway substrate 15*; FCRL4:* Fc receptor-like 4; *FCGR2B:* Fc gamma receptor IIb; *FOXO3:* Forkhead box O3*; FOXO4:* Forkhead box O4*; FOXP1:* Forkhead box P1*; FUS:* FUS RNA binding protein*; GRIP1:* Glutamate receptor interacting protein 1*; GPR34:* G protein-coupled receptor 34*; HMGA2:* High mobility group AT-hook 2*; HLF:* Hepatic leukemia factor*; HOXA10:* Homeobox A10*; HOXA13:* Homeobox A13; *HOXC11:* Homeobox C11*; HOXD13:* Homeobox D13*; ID4:* Inhibitor of DNA binding 4*; IGF2BP1:* Insulin-like growth factor 2 mRNA binding protein 1; *IRS4:* Insulin receptor substrate 4*; IRF4:* Interferon regulatory factor 4*; IRF8:* Interferon regulatory factor 8; *JAK2:* Janus kinase 2; *JAZF1:* JAZF zinc finger 1; *KAT6A:* Lysine acetyltransferase 6A*; KDM4C:* Lysine demethylase 4C*; KDSR:* 3-ketodihydrosphingosine reductase*; KMT2A:* Lysine methyltransferase 2A*; LCK:* LCK proto-oncogene, Src family tyrosine kinase*; LMO1:* LIM domain only 1*; LMO2:* LIM domain only 2*; LYL1:* Lymphoblastic leukemia associated hematopoiesis regulator 1*; LHX2:* LIM homeobox 2*; LHX4:* LIM homeobox 4; *MALT1:* MALT1 paracaspase*; MAFB:* MAF bZIP transcription factor B*; MAML2:* Mastermind-like transcriptional coactivator 2; *MAST2:* Microtubule associated serine/threonine kinase 2; *MECOM:* MDS1 and EVI1 complex locus*; MLLT10:* MLLT10 histone lysine methyltransferase DOT1L cofactor*; MLLT1:* MLLT1 super elongation complex subunit*; MLLT11:* MLLT11 transcription factor 7 cofactor*; MLLT3:* MLLT3 super elongation complex subunit*; MLLT4:* MLLT4, afadin*; MLLT6:* MLLT6 PHD finger containing*; MYB:* MYB proto-oncogene*; MYCN:* MYCN proto-oncogene, bHLH transcription factor*; MYC:* MYC proto-oncogene, bHLH transcription factor*; NFKB2:* Nuclear factor kappa B subunit 2*; NUMA1:* Nuclear mitotic apparatus protein 1*; NPM1:* Nucleophosmin 1*; NOTCH1:* Notch receptor 1*; NUP214:* Nucleoporin 214*; NUP98:* Nucleoporin 98*; PAFAH1B2:* Platelet activating factor acetylhydrolase 1b catalytic subunit 2*; PBX1:* PBX homeobox 1; *PICALM:* Phosphatidylinositol binding clathrin assembly protein*; PAX3:* Paired box 3*; PAX5:* Paired box 5*; PCSK7:* Proprotein convertase subtilisin/kexin type 7*; PRDM16:* PR/SET domain 16*; PRKAR1A:* Protein kinase cAMP-dependent type I regulatory subunit alpha*; PRRX1:* Paired related homeobox 1*; RUNX1:* RUNX family transcription factor 1*; RUNX1T1:* RUNX1 partner transcriptional co-repressor 1*; ROS1:* ROS proto-oncogene 1, receptor tyrosine kinase*; RET:* Ret proto-oncogene*; SLC45A3:* Solute carrier family 45 member 3*; SS18:* SS18 subunit of BAF chromatin remodeling complex*; SPIB:* Spi-B transcription factor*; SSX1:* SSX family member 1*; SSX2:* SSX family member 2*; TAL1:* TAL bHLH transcription factor *; TAL2:* TAL bHLH transcription factor 2*; TCF12: T*ranscription factor 12*; TCF3:* Transcription factor 3*; TFG:* Trafficking from ER to golgi regulator*; TERT:* Telomerase reverse transcriptase*; TLX3:* T-cell leukemia homeobox 3*; TRIM24:* Tripartite motif containing 24*; TPD52:* Tumor protein D5*2; TPM3:* Tropomyosin 3*; USP6:* Ubiquitin specific peptidase 6; *WHSC1:* Wolf-Hirschhorn syndrome candidate 1*; ZBTB16:* Zinc finger and BTB domain containing 16*; ZC3H12D:* Zinc finger CCCH-type containing 12D*; ZMYM2:* Zinc finger MYM-type containing 2*.*

**Supplementary Table 4:** **Characteristics of fusion genes using NGS and chromosome karyotyping.**

| **No. of sample** | **Group** | **Fusion gene 1** | **VAF (%)** | **Fusion gene 2** | **VAF (%)** | **Karyotype** |
| --- | --- | --- | --- | --- | --- | --- |
| YF00HFG00M000084 | Remission | *KMT2A->MLLT10* | 37.8 | *MLLT10->KMT2A* | 18.05 | 46,XY[20] |
| YF00HFG00M000080 | Remission | *PICALM->MLLT10* | 269.32 | *MLLT10->PICALM* | 32.48 | 47,XY,+4,?t(10;11)(p13;q14)[9]/47,XY,+4,?t(10;11)(p13;q14),?del(11)(p15)[8]/46,XY[6] |
| YF00HFG00M000043 | Refractory | *EIF4A1->ACTB* | 2.61 |  |  | 46,XY[23] |
| YF00HFG00M000061 | Refractory | *LMO1->RIC3* | 1.53 | *EIF4A1->CIC* | 0.84 | 47,XX,+11[3]/46,XX[10] |
| YF00HFG00M000059 | Refractory | *KMT2A->CTNND1* | 58.58 |  |  | complex karyotype |
| YF00HFG00M000057 | Refractory | *KMT2A->MLLT3* | 19.75 | *MLLT3->KMT2A* | 1.01 | 46,XY[20] |
| YF00HFG00M000039 | Refractory | *KMT2A->SEPT9* | 51.1 | *SEPT9->KMT2A* | 7.49 | 46,XY[20] |
| YF00HFG00M000056 | Refractory | *KMT2A->USP2* | 7.32 |  |  | 48,?del(9)(p11),?del(11)(q22),-17,-18,inc[cp25] |
| YF00HFG00M000071 | Refractory | *NSD1->NUP98* | 83.15 | *NUP98->NSD1* | 43.98 | 46,XY[20] |
| YF00HFG00M000081 | Remission | *NSD1->NUP98* | 56.56 | *NUP98->NSD1* | 26.7 | 46,XX[20] |
| YF00HFG00M000037 | Refractory | *NUP98->NSD1* | 23.55 | *B4GALT7->NUP98* | 8.38 | 47,XX,+6[14]/46,XX[6] |
| YF00HFG00M000049 | Refractory | *NUP98->NSD1* | 24.07 |  |  | 46,XY[25] |
| YF00HFG00M000079 | Refractory | *NUP98->NSD1* | 45.36 |  |  | 46,XY[20] |
| YF00HFG00M000060 | Refractory | *PRDM16->SKI* | 2.24 |  |  | 46,XY[23] |
| YF00HFG00M000051 | Relapse | *BRE->MECOM* | 6.02 |  |  | 46,XX[35]/hypodiploid [8](40–45) |
| YF00HFG00M000100 | Relapse | *CDK6->HOXA11-AS1* | 8.64 |  |  | 46,XY[20] |
| YF00HFG00M000092 | Relapse | *KMT2A-PTD* |  |  |  | 46,XY[20] |
| YF00HFG00M000098 | Relapse | *NUP98->GALNTL4* | 50.47 | *EPC1->GDI2* | 17.64 | complex karyotype |
| YF00HFG00M000108 | Relapse | *SEPT6->NKRF* | 1.57 |  |  | 46,XY[20] |
| YF00HFG00M000105 | Relapse | *KMT2A->MLLT3* | 24.07 |  |  | 46,XY[20] |
| YF00HFG00M000017 | Relapse | *KMT2A->MLLT10* | 22 | *MLLT10->SNX32* | 2.95 | 46,XY,t(10;11)(p13;q13)[20] |

*B4GALT7*-*NUP98*: Beta-1,4-galactosyltransferase 7 - nucleoporin 98; *BRE*-*MECOM*: Brain and reproductive organ-expressed-MDS1 and EVI1 Complex Locus; *CDK6*-*HOXA11-AS1*: Cyclin-dependent kinase 6 - HOXA11 antisense RNA 1; *EIF4A1*-*ACTB*: Eukaryotic translation initiation factor 4A1-actin beta; *EIF4A1*-*CIC*: Eukaryotic translation initiation factor 4A1-capicua transcriptional repressor; *EPC1*-*GDI2*: Enhancer of polycomb homolog 1-GDP dissociation inhibitor 2; *KMT2A*-*CTNND1*: Lysine methyltransferase 2A-Catenin delta 1 fusion; *KMT2A*-*MLLT10*: Lysine methyltransferase 2A-mixed-lineage leukemia translocated to 10; *KMT2A*-*MLLT3*: Lysine methyltransferase 2A-mixed-lineage leukemia translocated to 3; *KMT2A*-*PTD*: Lysine methyltransferase 2A partial tandem duplication; *KMT2A*-*SEPT9*: Lysine methyltransferase 2A-septin 9; *KMT2A*-*USP2*: Lysine Methyltransferase 2A-ubiquitin specific peptidase *2*; *LMO1*-*RIC3*: LIM domain only 1-RIC3 cholinergic receptor chaperone; *MLLT10*-*KMT2A*: Mixed-lineage leukemia translocated to 10-lysine methyltransferase 2A ; *MLLT10*-*PICALM*: Mixed-lineage leukemia translocated to 10-phosphatidylinositol binding clathrin assembly protein; *MLLT10*-*SNX32*: Mixed-lineage leukemia translocated to 10-sorting Nexin 32; *MLLT3*-*KMT2A*: Mixed-lineage leukemia translocated to 3-lysine methyltransferase 2A ; *NSD1*-*NUP98*: Nuclear receptor binding SET domain protein 1-nucleoporin 98; *NUP98*-*GALNTL4*: Nucleoporin 98 - polypeptide n-acetylgalactosaminyltransferase like 4; *NUP98*-*NSD1*: Nucleoporin 98-nuclear receptor binding SET domain protein 1; *PICALM*-*MLLT10*: Phosphatidylinositol binding clathrin assembly protein-mixed-lineage leukemia translocated to 10; *PRDM16*-*SKI*: PR domain containing 16-SKI proto-oncogene; *SEPT6*-*NKRF*: Septin 6-NFKB repressing factor; *SEPT9*-*KMT2A*: Septin 9-Lysine Methyltransferase 2; VAF: Variant allele frequency.
